# TFAB002s, novel CD20-targeting T cell-dependent bispecific Fab-FabCH3 antibodies, exhibit potent antitumor efficacy against malignant B-cell lymphoma

**DOI:** 10.1371/journal.pone.0310889

**Published:** 2024-09-25

**Authors:** Qinghong Li, Kunming Zhang, Yao Yu, Zeng Yu, Jingyi Xu, Wenyan Shen, Lin Zhang, Aidong Qu, Hongyuan Liang

**Affiliations:** No.1 Research Laboratory, Shanghai Institute of Biological Products Co., Ltd., Shanghai, China; European Institute of Oncology, ITALY

## Abstract

B-cell lymphoma, clinically, comprises a heterogeneous group of malignancies that encompass various subtypes. CD20 is an optimal target for therapeutic antibodies in B-cell lymphoma immunotherapy since approximately 90% of B-cell malignancies typically exhibit CD20 expression on their surface, while its presence is limited in normal tissues. In this study, we have developed a series of novel non-IgG-like T cell-dependent bispecific antibodies by constructing Fab-FabCH3, referred to as Tandem Antigen-binding Fragment 002 (TFAB002), which specifically target CD20 for the treatment of malignant B-cell lymphoma. TFAB002s display strong binding affinity with CD20 and moderate binding affinity with CD3, thereby triggering target-specific T-cell activation, cytokine release, and tumor cell lysis *in vitro*. Furthermore, TFAB002s exhibit potent cytotoxicity against B-cell malignancies that express varying levels of CD20. Besides, the TFAB002s show potent pharmacodynamic activity *in vivo* in the WIL2-S cells CDX mouse model. Collectively, these results underscore the potential of TFAB002s as a highly promising therapeutic approach for selectively depleting CD20-positive B cells, thereby warranting further clinical evaluation as a viable treatment option for CD20-expressing B-cell malignancies.

## Introduction

B-cell lymphoma (BCL) is a neoplastic proliferation of B-cells that manifests as solid tumors, including Hodgkin’s and non-Hodgkin’s lymphomas. It is one of the most prevalent malignancies worldwide, recognized as a member of the ‘top 10 malignant tumors’, constituting approximately 5% of all cancer cases [1, 2]. Malignant B cells often exhibit markers as CD19, CD20, CD22, CD23, CD30, CD38, paired box 5 (PAX5), B-cell lymphoma-2 (BCL-2), B-cell maturation antigen (BCMA), lymphoid enhancer-binding factor 1 (LEF1), and others [3].

CD20, a member of the membrane-spanning 4-domain family, subfamily A (MS4A) family, is a nonglycosylated phosphoprotein that forms a tetraspanin membrane-bound protein and serves as a specific marker for B cells [4]. CD20 expression is typically observed on the surface of approximately 90% of B-cell malignancies while its presence is limited in normal tissues [2]. Specifically, CD20 can be detected on pre-B and mature B lymphocytes but not on hematopoietic stem cells, post-B cells, normal plasma cells, or other non-malignant tissues [5]. This unique feature makes CD20 an optimal target for therapeutic antibodies in tumor immunotherapy.

Rituximab, the first monoclonal antibody targeting CD20 approved for therapeutic application, has shown efficacy in over 50% of patients with relapsed or refractory CD20-positive follicular non-Hodgkin’s lymphoma at standard weekly dosing [6]. However, it does not confer a curative effect. Moreover, its effectiveness is diminished in other subtypes of CD20-positive lymphoma and cases of retreatment, even when CD20 expression is still present. Therefore, the binding of rituximab to CD20 alone is insufficient to eliminate lymphoma cells completely, indicating the existence of resistance mechanisms [7, 8].

To overcome this type of resistance, we are exploring alternative mechanisms of action (MoA), such as T cell-dependent bispecific antibodies (TDBs). Redirecting T-cell activity through TDBs against tumor cells, regardless of their T-cell receptor (TCR) specificity, represents a potent therapeutic strategy for cancer treatment [9]. This approach relies on the recognition of tumor cell surface antigens and simultaneous binding to the CD3 epsilon chain (CD3ε) within the TCR complex. Consequently, it elicits robust T-cell activation characterized by the release of cytotoxic molecules, cytokines, and chemokines, as well as induction of T-cell proliferation [10]. Till recently, the Food and Drug Administration (FDA) has approved three IgG-like TDBs, namely Mosunetuzumab and Glofitamab by Roche, as well as Epcoritamab by Genmab, which exhibit distinct structural features and varying antigen-binding titers. However, several reported modalities that employ T-cell recruitment via TDBs encounter limitations due to unfavorable pharmacokinetics (PK), potential concerns about immunogenicity, and manufacturing challenges [11, 12]. In this study, we aim to address these existing limitations of TDBs by designing a novel Fab-FabCH3 structure, distinct from bispecific T cell engagers (BiTE), F(ab)_2,_ and previously mentioned IgG-like architectures. We anticipate that the novel Fab-FabCH3 architecture can (1) incorporate the CH3 domain using knobs-into-holes (KiH) technology to facilitate antibody binding to FcRn, thereby extending antibody half-life; (2) minimize immunogenicity of the new structure by avoiding introduction of any novel point mutations, except for the mutation associated with KiH technology; (3) address light-heavy chain mismatch by utilizing CH1 and CH3 as distinct heavy chains for diverse antigen-binding domains.

This article highlights a series of innovative molecular characteristics of CD20 TDBs and provides insights into intriguing aspects related to the biological activity of these molecules, including their binding affinity with CD3 and CD20 antigens, cell-redirection activity, T cell activation activity, as well as cytotoxicity *in vitro* and *in vivo*.

## Materials and methods

### Cell cultures

WIL2-S cell line was purchased from the American Type Collection (ATCC cat. #CRL-8885, RRID: CVCL_3809). JeKo-1 cell line was purchased from the National Collection of Authenticated Cell Cultures (CCRID cat. #3101HUMTCHu194, RRID: CVCL_1865). Jurkat, Clone E6-1 cell line was purchased from the National Collection of Authenticated Cell Cultures (CCRID cat. #3101HUMSCSP513, RRID: CVCL_0367). CHO-K1 cell line was purchased from the National Collection of Authenticated Cell Cultures (CCRID cat. #3101HAMSCSP507, RRID: CVCL_0214). Jurkat-NFAT cell line was purchased from Novoprotein Scientific Inc. (cat. #XCC20, RRID: CVCL_E2U5). H_FCGR2B(CD32B) CHO-K1 cell line was purchased from Genomeditech Co., Ltd. (cat. #GM-C16925, RRID: CVCL_E2SY). Cell lines were cultured according to instructions provided by the ATCC. Jurkat-NFAT cells were cultured in RPMI-1640 media (cat. #A10491-01, Gibco) containing 10% fetal bovine serum (FBS) (cat. #abs972, Absin) and 100μg/ml Hygromycin B (cat. #10687–010, Gibco). H_FCGR2B(CD32B) CHO-K1 cells were cultured in F12K (cat. #21127022, Gibco) containing 10% FBS,100 μg/ml Hygromycin B and 4 μg/ml Puromycin (cat. #A11138-03, Gibco). CD3^+^ T cells were purchased from MILESTONE (Shanghai, China). PBMC (lot. #A10S092036) cells were purchased from MILESTONE (Shanghai, China).

### Generation of TFAB002s

The TFAB002 antibodies were designed as a tandem Fab-FabCH3 format, but one of the CH1-CL domains was replaced by the CH3 domain using KiH technology to reduce mismatching. The TFAB002s construct consisted of three chains: a standard anti-CD20 light chain, an engineered anti-CD3 heavy or light chain containing only VH or VL and CH3 domains, and a fusion chain consisting of an anti-CD20 heavy chain in Fab format and an engineered anti-CD3 light or heavy chain with the CL or CH1 domain replaced by CH3 domain. The antibodies were purified using TOYOPEARL AF-rProteinL-650F (cat. #0023488, TOSOH) according to the manufacturer’s protocols.

### Fluorescence-activated cell sorting (FACS) assay

The binding activity of TFAB002s to CD20-expressing WIL2-S cells, CD3-expressing Jurkat cells, FcRn-expressing H_FCGR2B CHO-K1 cells, parent cell line CHO-K1 cells, and the CD20 expression level on WIL2-S and JeKo-1 cells were evaluated. Briefly, cells were incubated with serial dilutions of TFAB002s, Rituximab (lot. #RX220101; SIBP), or isotype hIgG1 (lot. #CL230901; SIBP) for 1h at 4°C, washed with phosphate-buffered saline (PBS) containing 2% FBS and stained with a secondary Alexa Fluor^™^ 488 goat anti-human IgG (H+L) (cat. #A11013, Invitrogen) or Mouse anti-Human Kappa Light Chain Secondary Antibody, APC (cat. #MH10515, Invitrogen) for 45min at 4°C. Subsequently, the cells were washed and resuspended in PBS with FBS for flow cytometry analysis, and the mean fluorescence intensity was calculated. The concentration-response curves and EC_50_ values were fitted and calculated using GraphPad Prism version 9.5.1 for Windows, GraphPad Software, Boston, Massachusetts USA, www.graphpad.com.

### Cell redirection *in vitro* imaging

The CD3-positive Jurkat cells were labeled with CellTracker^™^ Blue 7-amino-4-chloromethlcoumarin (CMAC) (cat. #C2110, Invitrogen) or carboxyfluorescein diacetate succinimidyl ester (CFSE) (cat. #565082, BD Biosciences). The CD20-positive WIL2-S cells were labeled with CellTracker^™^ Deep Red (cat. #C34565, Invitrogen). Antibodies were conjugated using the fluorescein labeling kit-NH_2_ (cat. #LK01, Dojindo), following the manufacturer’s instructions. Subsequently, the labeled Jurkat and WIL2-S cells were mixed at an effector-to-target ratio of 3:1 and treated with TFAB002s at concentrations of 300nM or 50nM for 1h at 4°C followed by an additional hour at 37°C. T-cell redirection was visualized through fluorescence imaging using a high-content analysis system (PerkinElmer). The resulting data was analyzed using Cellprofiler [13].

### Jurkat-NFAT luciferase reporter cell assay

The activation of CD3 downstream signaling was observed at 6h of co-culture of Jurkat-NFAT luciferase receptor and WIL2-S cells or JeKo-1 cells in the presence of serial dilutions of TFAB002s. Relative luminescence units were quantified using the Bio-Glo luciferase assay reagent (cat. #G7940, Promega) with a SpectraMax M5 reader (Molecular Devices). Concentration-response curves and EC_50_ values were fitted and calculated using GraphPad Prism version 9.5.1 for Windows, GraphPad Software, Boston, Massachusetts USA, www.graphpad.com.

### Redirected T-cell cytotoxicity

Redirected T-cell cytotoxicity was assessed by detecting dead cells using 7-amino-actinomycin D (7-AAD) (cat. #640922, BioLegend) by flow cytometry. CD3^+^ T cells were co-incubated with target cells labeled with CFSE at an effector-to-target ratio of 3:1 and serial dilutions of TFAB002s for 36h in 6-well plates. Dead target cells were gated as PerCP^+^FITC^+^ for killing assays.

### Cytokine release assay

The CD3+ T cells were co-cultured with WIL2-S cells (effector: target ratio = 3:1) and TFAB002s at concentrations of 10nM for 36h in a 6-well plate. The culture medium was collected, and the supernatants were utilized for cytokine release detection using the HU Th1/Th2 Panel (8-plex) w/VbP V02 (cat. #741030, BioLegend), following the manufacturer’s instructions. A standard curve was generated with eight concentration gradients ranging from 0pg/ml to 10000pg/ml, employing a four-fold dilution series. Data acquisition was performed using NovoCyte Advanteon (Agilent), and analysis was conducted utilizing the LEGENDplex^™^ Data Analysis Software Suite, an online cloud-based program, 2019 Qognit, Inc., https://legendplex.qognit.com/workflow.

### Xenograft mouse model

Female C-NKG mice, 39 days of age, were obtained from Cyagen (Suzhou, China) and maintained under pathogen-free conditions at the Experimental Animal Center of Shanghai Institute of Biological Products Co., Ltd. After 7 days of acclimatization, each mouse was implanted with 1 × 10^7^ WIL2-S cells mixed with matrigel at the right flank. When tumors reached the desired volume, mice were grouped randomly based on administered different doses of TFAB002s or PBS intratumorally twice a week along with PBMC intratumorally once a week. The tumor volumes and body weights of the mice were recorded twice per week. The tumor volume was measured with calipers and calculated according to the formula: 1/2 × maximum length of major axis (L) and maximum length of minor axis (W)^2^. Mice were euthanized humanely when the observation no longer made sense. The animal experiments were approved by the Animal Use and Care Committee of Shanghai Institute of Biological Products Co., Ltd.

### Statistical analysis

The data was presented as mean, mean ± standard derivation, or mean + standard derivation. The difference between multiple groups was compared using One-way ANOVA or Two-way ANOVA followed by Tukey’s multiple comparison test, respectively. A significance level of *p*<0.05 was considered statistically significant.

## Results

### TFAB002s exhibited distinct binding affinities towards CD20 and CD3

**Fig 1:**
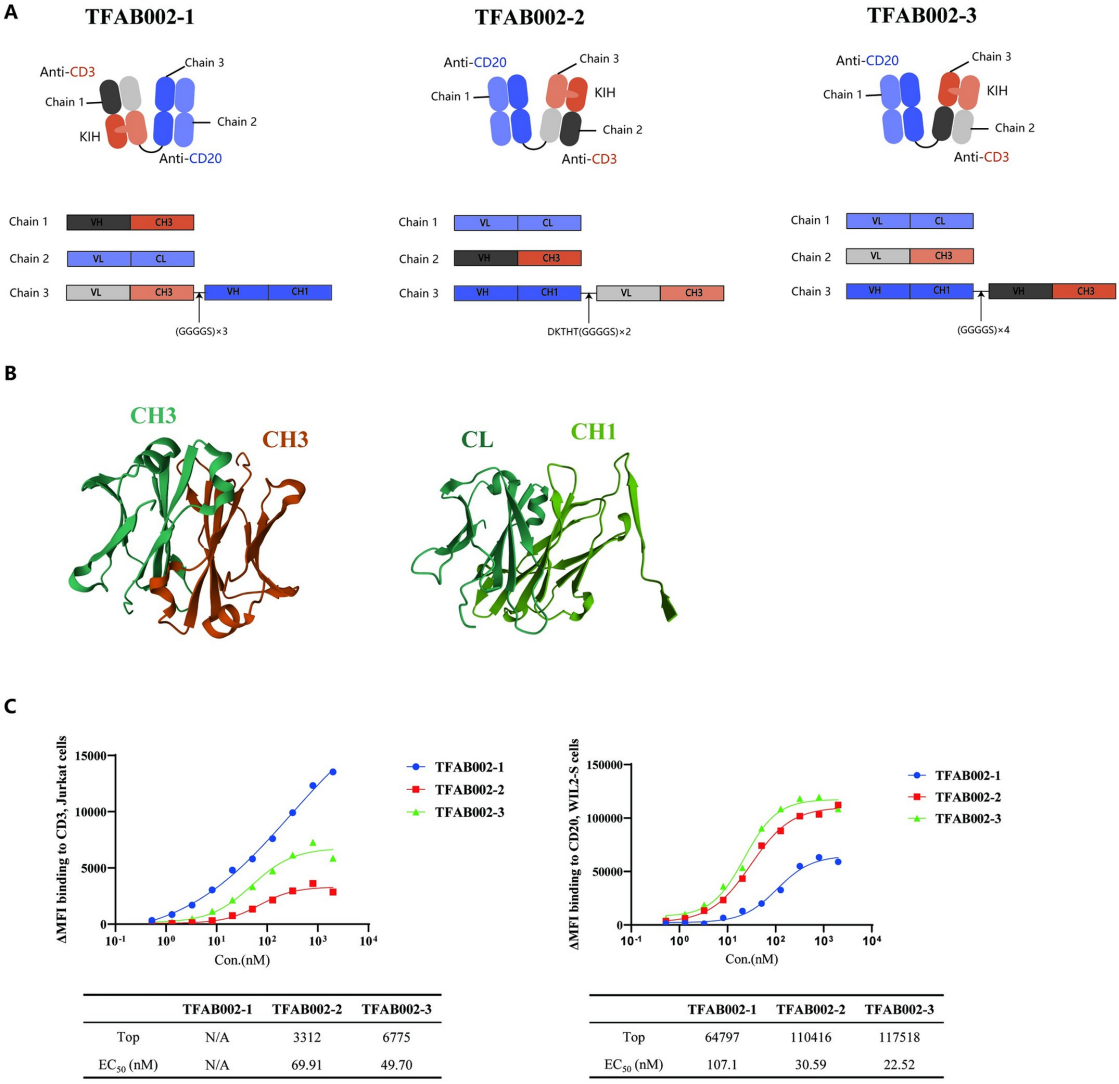
TFAB002s exhibited significant binding affinity towards CD20 and CD3. (A) Schematic representation of TFAB002s, which consist of three chains that specifically target CD20 and CD3ε in a monovalent binding mode. VH, variable domain of heavy chain; VL, variable domain of light chain; CL, constant domain of light chain; CH, constant domain of heavy chain. (B) Presentation of human IgG1 CH3 domains (PDB entry: 2WAH) and IgG1 CH1-CL domains (PDB entry: 4F33). (C) FACS was performed to assess TFAB002s’ binding ability with CD3 on Jurkat cells and CD20 on WIL2-S cells. N/A means that a reasonable result is not fitted based on the experimental data.

TFAB002s are a series of Fab-FabCH3 format bsAbs composed of one Fab targeting CD20 and one engineered Fab targeting CD3 with the CH1-CL domain replaced by the CH3 domain (Fig 1A). This design concept was inspired by literature findings that highlighted the high homology between an IgG1 CH3 domain pair and a CH1-CL heterodimer (Fig 1B) [14]. Moreover, linkers of varying lengths were employed to enhance the formation of immune synapses and improve antigen-binding affinity. The N-terminal CD3-targeting TFAB002-1 consists of a linker which is (GGGGS)_3_. Similarly, the N-terminal CD20-targeting TFAB002-2 contains a linker which is DKTHT(GGGGS)_2_, whereas the linker of the TFAB002-3 is modified into (GGGGS)_4_ based on TFAB002-2. The production levels of TFAB002-1 and TFAB002-2 were achieved at 35 mg/L, while the production level of TFAB002-3 was attained at 55mg/L. TFAB002s were detected by size-exclusion chromatography (SEC), the main peak areas of TFAB002-1, TFAB002-2, and TFAB002-3 were 94.28%, 93.02%, and 96.76% respectively. The binding affinity of TFAB002s to CD20 was assessed via FACS using CD20-positive WIL2-S cells, while the binding affinity to CD3 was evaluated using CD3-positive Jurkat cells. As depicted in Fig 1C, TFAB002s bound to both WIL2-S and Jurkat cells. Notably, TFAB002-1 displayed an enhanced binding affinity with CD3. Similarly, TFAB002-2 and TFAB002-3, which had CD20 antigen-binding sites on the N-terminal, showed higher affinity towards CD20. Moreover, we observed that TFAB002-3 with a longer linker had a superior binding affinity towards CD3 compared to TFAB002-2. Collectively, these findings indicated steric hindrance at the middle chains’ binding site in this form of bsAb and emphasized how linker length impacted its overall binding affinity.

### TFAB002s facilitated T-cell redirection

**Fig 2:**
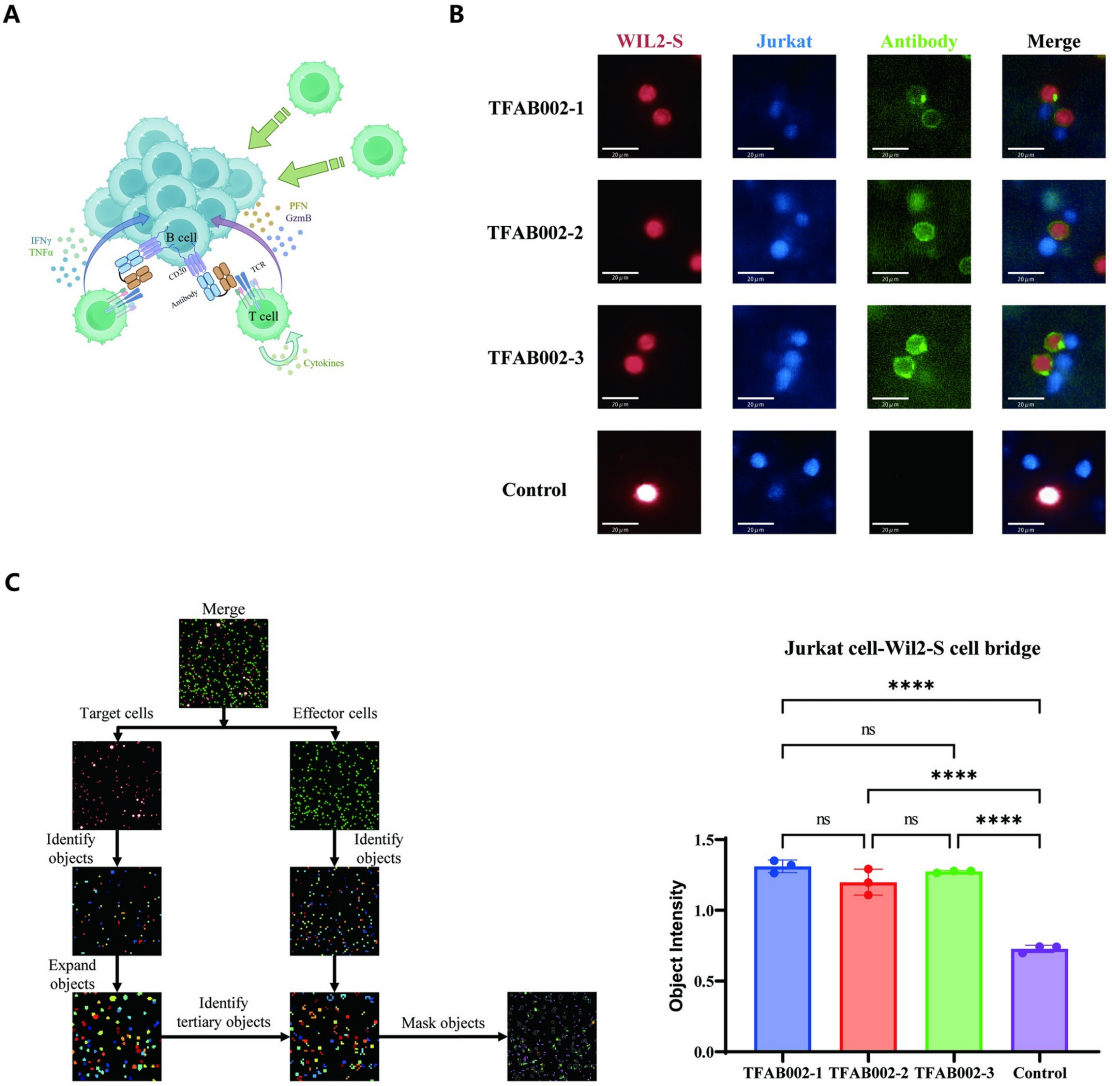
TFAB002s redirected CD3-expression Jurkat cells to CD20-expression WIL2-S cells. (A) Schematic representation of the mechanisms that TFAB002s recruit T cells to tumor cells surroundings. By Figdraw. (B) *In vitro* visualization of TFAB002s (green) during interactions between CD3^+^ Jurkat cells (blue) and CD20^+^ WIL2-S cells (red). The image array highlighted the localization of TFAB002s at the immune synapse at the cell membrane contact. Scale bar, 20μm. (C) The graph on the left illustrated the analysis process using Cellprofiler. The graph on the right presented a quantification of TFAB002s’ cell-redirection activity. Error bars represent the standard deviation from three independent experiments means. A Tukey one-way ANOVA test for mask objects in treated groups compared to control demonstrated statistical significance (****p<0.0001, adjusted for multiple hypotheses).

We investigated the mechanism underlying the cytotoxicity of bsAbs TFAB002s targeting CD3 and CD20 against B lymphoma cells (Fig 2A). Live-cell imaging of WIL2-S cells, Jurkat cells, and fluorescently labeled TFAB002s *in vitro* cocultures was used to visualize antibody binding (Fig 2B). TFAB002s significantly increased the tumor/T cell immune synapse area, indicating enhanced interaction between tumor cells and T cells that correlated with synaptic localization of TFAB002s. Notably, in the presence of 50nM TFAB002s (Fig 2C), CD3-positive Jurkat cells were observed to assemble around CD20-expressing WIL2-S cells. These findings unequivocally demonstrated that TFAB002s recruited T cells to target CD20-expression on WIL2-S cells.

### TFAB002s promoted the T-cell activation and the release of inflammatory cytokines dependent on the presence of CD20-positive target cells

**Fig 3:**
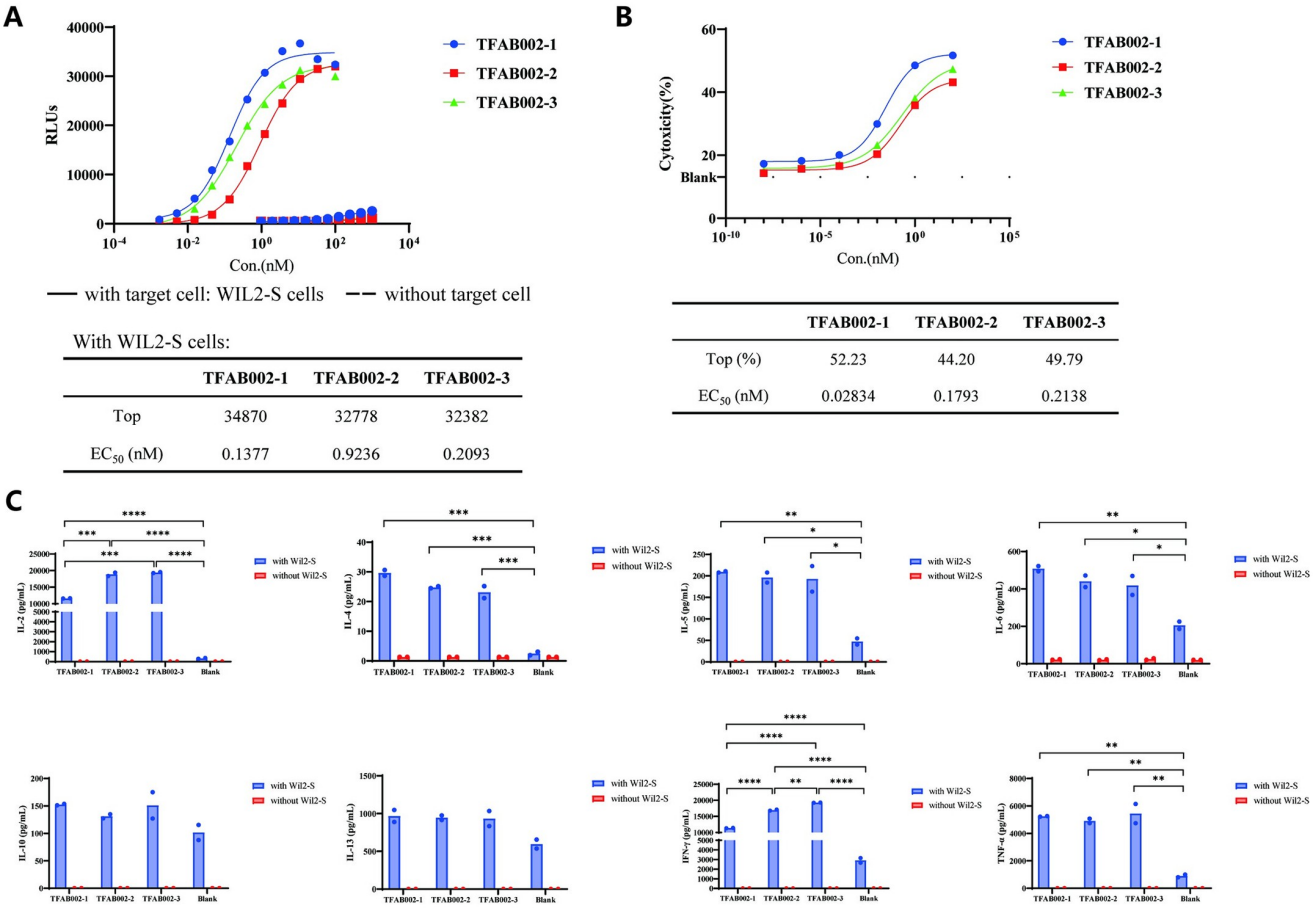
TFAB002s activated T cells and promoted T-cell mediated killing of lymphoma B cells. (A) T-cell activation was measured through the NFAT luciferase reporter assay. (B) The cytotoxicity against CD20-expressing WIL2-S cells induced by TFAB002s was evaluated by quantifying dead cells with 7-AAD. (C) The cytokine release induced by TFAB002s in co-culture with T cells and WIL2-S cells or in the culture of T cells. A Tukey one-way ANOVA test for mask objects in treated groups compared to control demonstrated statistical significance (****p<0.0001, ***p<0.001, **p<0.01, *p<0.05, adjusted for multiple hypotheses).

To evaluate the activation of T cells by TFAB002s, co-cultures were established using WIL2-S cells and Jurkat cells that stably express the NFAT-luciferase reporter gene. Subsequently, the downstream signaling of CD3 was quantified. As shown in Fig 3A, TFAB002s effectively activated T cells in the presence of CD20-expressing WIL2-S cells. TFAB002s caused a cross-linking interaction between WIL2-S and CD3^+^ T cells, leading to the lysis of WIL2-S cells (Fig 3B). We also measured cytokine production by T cells when cocultured with target cells in the presence of TFAB002s. Remarkably, TFAB002s significantly upregulated the release of cytokines associated with cellular immunity, such as interleukin (IL)-2, interferon-γ (IFN-γ), and tumor necrosis factor-α (TNF-α), which ultimately mediated cytotoxicity against WIL2-S cells (Fig 3C). In contrast, a control assay without target cells showed no detectable release of cytokines. The concentration of IL-6, a cytokine associated with inflammation, exhibited statistically significant elevations, while the statistical significance was not observed in the differences in concentration of IL-10, a cytokine associated with immunosuppression (Fig 3C). Collectively, TFAB002s exhibited conditional agonism on T cell function dependent on target cell presence (Fig 3C). Notably, among all variants tested for their potential impact on cytotoxicity and cytokine release syndrome (CRS), TFAB002-3 displayed enhanced T-cell activation and cytotoxicity along with improved safety *in vitro*.

**Fig 4:**
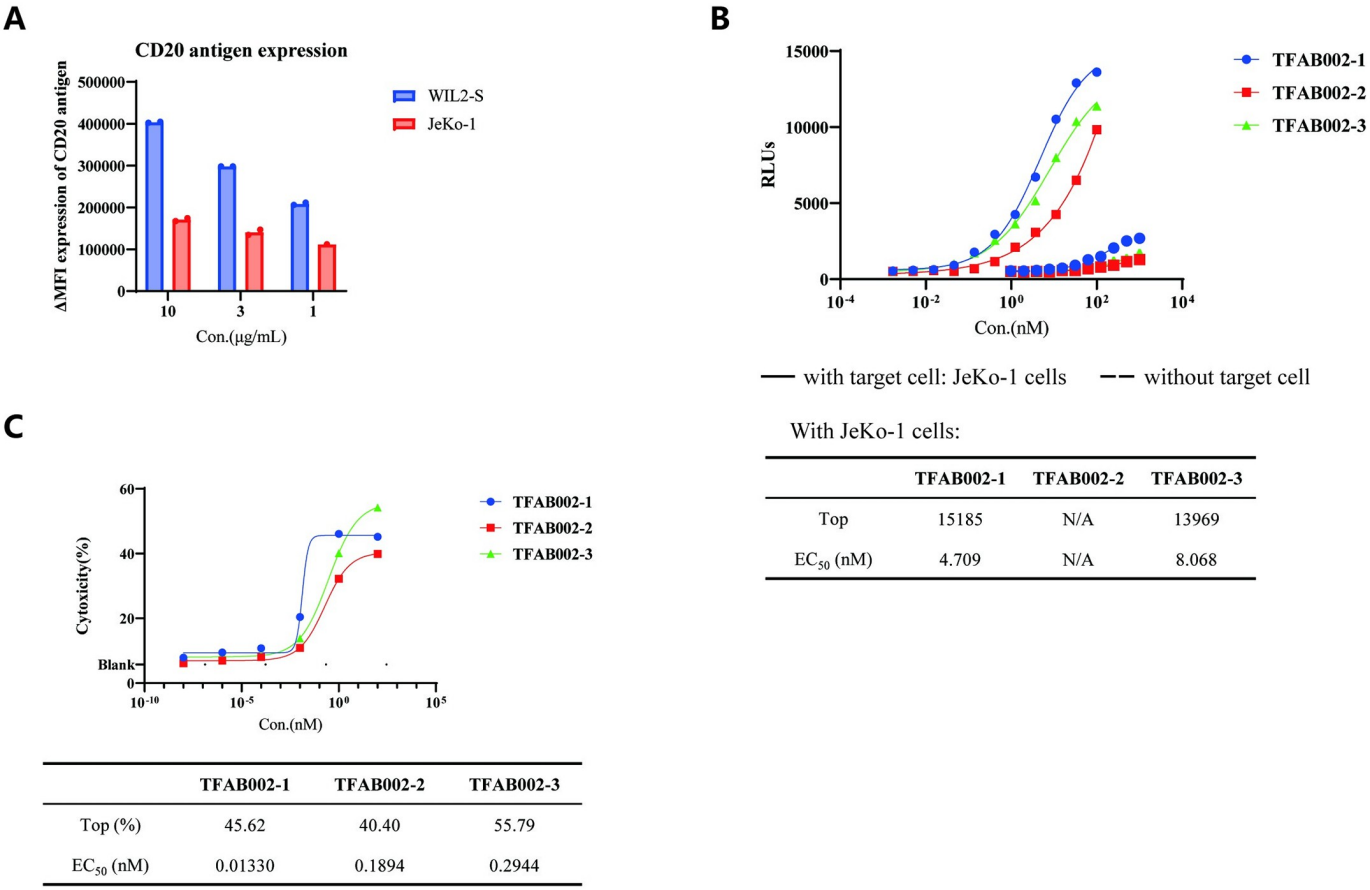
TFAB002s’ biological activity was observed to be similar across different tumor cell lines. (A) The expression level of CD20 on tumor cell lines was compared using FACS analysis. (B) T cell activation was assessed by measuring NFAT luciferase reporter assay. N/A means that a reasonable result is not fitted based on the experimental data. (C) The cytotoxicity of TFAB002s against CD20-expressing JeKo-1 cells was evaluated by quantifying dead cells with 7-AAD.

Besides, we validated these findings by performing the same assay using an alternative target cell. We assessed the CD20-expression levels on different tumor cell lines (WIL2-S cells, a B lymphocyte cell line, and JeKo-1 cells, a mantle cell lymphoma (MCL) cell line) using the anti-CD20 antibody Rituximab (Fig 4A). As illustrated in Fig 4B and 4C, these results demonstrated consistent trends with the previous observations. Moreover, when the surface expression of CD20 on JeKo-1 cells was relatively low, TFAB002-3 maintained comparable potent cytotoxicity effects in the cell-killing assay as described above. These outcomes provided compelling evidence that TFAB002s effectively engaged T cells and elicited cytotoxicity against CD20-positive tumor cells.

### TFAB002s demonstrated antitumor activity *in vivo*

**Fig 5:**
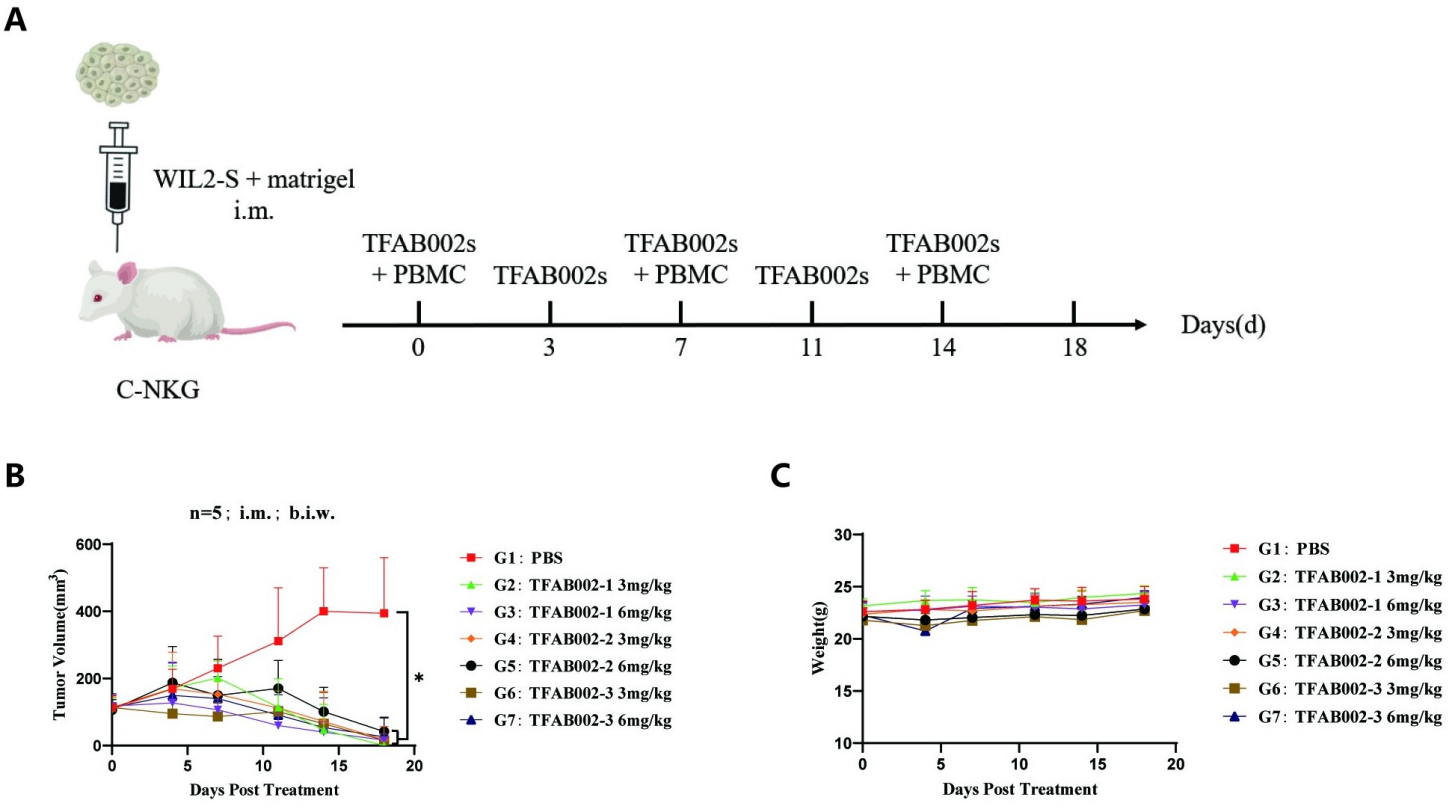
TFAB002s exhibited favorable safety and efficacy *in vivo*. (A) Schematic diagram of efficacy experiment design *in vivo*. (B) Alterations in tumor volume were observed in the WIL2-S cell CDX model mice. Error bars represent the standard deviation from five independent experiments means. A Tukey two-way ANOVA test for curves in treated groups compared to the control group demonstrated statistical significance (*p<0.05, adjusted for multiple hypotheses). (C) Body weight change curves of WIL2-S cell CDX model mice. Error bars represent the standard deviation from five independent experiments means.

To further evaluate the activity of TFAB002s *in vivo*, we established a WIL2-S cell C-NKG mouse CDX model and administered TFAB002s via intratumoral injection (Fig 5A). A significant reduction in tumor size was observed for all three strains of TFAB002s at doses of 3 mg/kg and 6 mg/kg, indicating a pronounced inhibitory effect on WIL2-S transplanted tumors (Fig 5B). Statistical analysis using Two-way ANOVA revealed a significant difference in tumor volume between the experimental group and the control group. Moreover, the mice in all experimental groups exhibited consistent weight comparable to those of the control group, thereby demonstrating the excellent safety profile of TFAB002s experiments *in vivo* (Fig 5C).

### TFAB002s exhibited binding affinity towards FcRn

**Fig 6:**
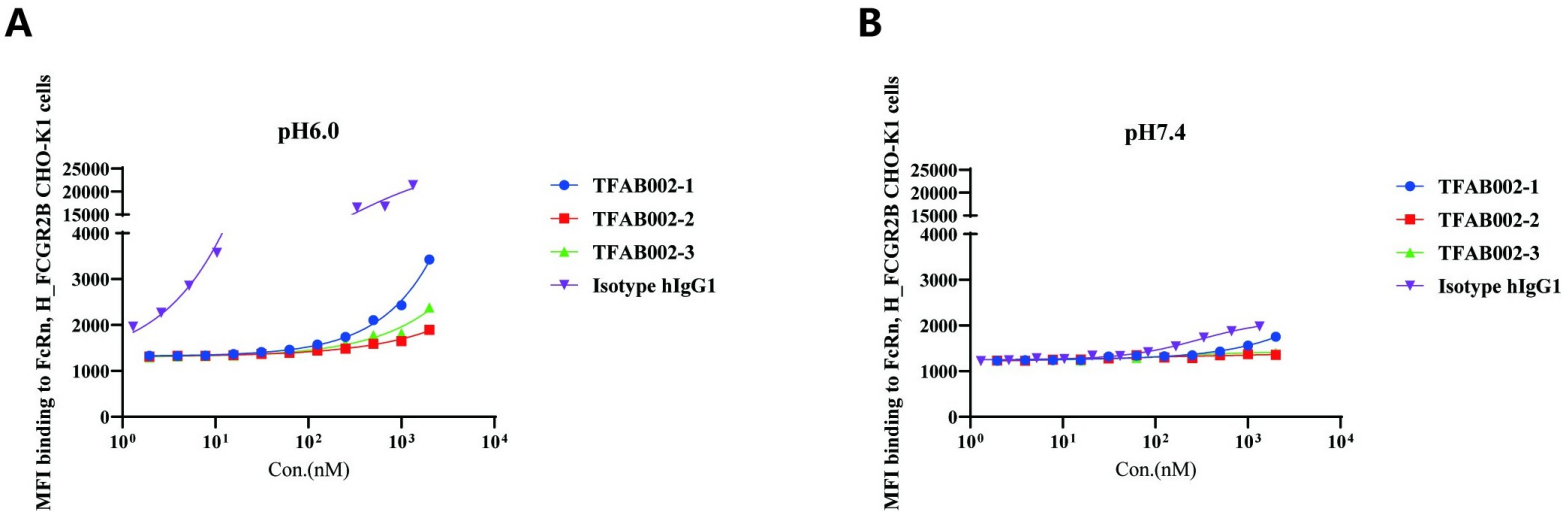
TFAB002s exhibited binding affinity towards FcRn. FACS was performed to assess TFAB002s’ binding ability with FcRn on H_FCGR2B CHO-K1 cells (A) at pH 6.0 and (B) at pH 7.4.

The primary drawback of non-IgG-like bispecific antibodies, such as the BiTE-structured Blinatumomab, lies in their remarkably limited half-life. This limitation arises not only due to the lower molecular weight of the bispecific antibodies (less than 60kDa), which leads to rapid renal clearance but also because non-IgG-like bispecific antibodies cannot bind with FcRn, thereby impeding their circulation within the bloodstream. This innovative “Fab-FabCH3” antibody, demonstrates a relative molecular mass of 100kDa and incorporates a CH3 domain as a replacement for the CH1-CL domain. The binding affinity of TFAB002s to FcRn was evaluated using H_FCGR2B CHO-K1 cells through FACS analysis (Fig 6 and S1 Fig). The findings revealed that the incorporation of a single CH3 domain preserved the weak pH-dependent interaction between the antibody and FcRn, thereby providing a promising avenue for future investigations into enhancing antibody binding activity to FcRn and extending antibody half-life.

## Discussion

Despite significant progress in the treatment of BCL over the past two decades, the recurrence rate among BCL patients remains unacceptably high, exceeding 50% even after various therapeutic interventions such as high-dose chemotherapy and Rituximab [15]. Notably, bispecific antibody-based immunotherapies have shown promising efficacy in clinical trials for BCL [16]. CD3-bispecific molecules have demonstrated exceptional preclinical potency when engaged with diverse tumor-associated antigens [17]. In this study, we developed TFAB002s, a series novel Fab-FabCH3 format of bsAb binding both CD20 and CD3 effectively, facilitating T cell activation and inducing CD20-targeted killing of lymphoma B cells *in vitro* and *in vivo*.

TFAB002s is a novel series of Fab-FabCH3 antibodies specifically targeting CD20-expressing tumor cells, different from previously KiH IgG or Duobody antibodies such as Mosunetuzumab and Epcoritamab. The design of TDBs plays a crucial role in determining their efficacy in tumor cell elimination, biodistribution, tumor penetration, selectivity for tumor cells over normal cells, immunogenicity, and pharmacokinetic properties [18]. These factors determine the clinical effectiveness and safety of TDB [19]. Notable features of TFAB002s include (i) a head-to-tail fusion via a flexible linker between the CD20- and CD3ε-binding Fab domains, ensuring potent binding avidity towards tumor cells while limiting affinity towards T cells, (ii) conditional T-cell activation dependent on the presence of target cells, (iii) closer immune synapse formation, and (iv) an engineered CH3 domain with knob-into-hole technology instead of the CH1-CL domain, which confers an extended half-life but abolishes its binding to complement component (C1q) and to Fc gamma receptors (FcγR).

Compared to conventional IgG format antibodies, TFAB002s have a lower molecular weight due to the deletion of a segment in the CH1 and CH2 domains. This leads to an incomplete Fc segment structure, inhibiting FcγR-mediated co-activation of innate immune effector cells, such as natural killer (NK) cells, monocytes/macrophages, and neutrophils [10, 20, 21]. Consequently, TFAB002s lack complement-dependent cellular cytotoxicity (CDC) and antibody-dependent cellular cytotoxicity (ADCC) activity; they only engage T cells as immune effector cells and avoid non-specific activation [22, 23]. Besides, the lower molecular weight can enhance tumor penetration. However, the stability of this new structure needs to be further optimized in the follow-up research and development process. Though these bsAbs are non-IgG format, we do not introduce new amino acid mutations, so the immunogenicity is manageable [24]. The introduction of a CH3 domain to replace one CH1-CL domain partially preserves the pH-dependent binding of the antibody to FcRn, which can consequently extend the antibodies’ half-life [25]. After TFAB002s in this novel Fab-FabCH3 format bind to cells, the distance between T cells and target cells is about only one Fab domain, which is more conducive to T-cell activation [26].

We selected TFAB002s with low-affinity CD3ε binding to limit non-specific immune activation and reduce associated side effects of TDBs, thereby conferring monovalent binding of TFAB002s to CD3ε [27]. This property, combined with the elimination of ADCC and CDC, ensures a high level of specificity as the immune synapse formed by simultaneous binding of both CD20 and CD3-directed domains. This property is essential for optimal T-cell activation and tumor cell lysis [28]. Based on this, we contended that the structures of TFAB002-2 or -3 had superior characteristics compared to TFAB002-1. The binding activity to CD3 and T-cell activation can be modulated by adjusting the length of the linker.

Uncontrolled systemic cytokine release is a common major challenge for T cell-engaging immunotherapies, irrespective of the target or treatment modality [29]. Urgent attention is required to optimize the toxicity management of anti-CD3 antibodies to maximize the therapeutic index of T-cell-engaged therapy. The rationale behind TFAB002s is to mitigate cytokine release while maintaining its efficacy compared with other CD20/CD3 bsAb [16]. We assessed the cytotoxicity and cytokine release induced by TFAB002s in a CD20-dependent manner using a lymphoma B cell line. Compared to the previously reported Duobody-CD3/CD20 mediated T cells killing JeKo-1 cells, the maximum efficacy in terms of lethality reaches approximately 60%, and the potency of antibody-mediated T-cell cytotoxicity is comparable; however, the EC_50_ values for TFAB002s are higher, approximately an order of magnitude greater than that observed for Duobody-CD3/CD20 [30]. In another study, researchers utilized Toledo, U2932, and Z-138 as target cells to concurrently compare the cytotoxic effects *in vitro* of three CD20-TDB antibodies with distinct structures and titers resembling IgG-like molecules, including 2:1 CD20-TDB, 1:1 OA CD20-TDB, and1:1 IgG CD20-TDB. Except when U2932 was employed as the target cell, the observed killing efficacy was only approximately 30%, and the cytotoxicity potency against the other two target cells still reached 50%. Nevertheless, when compared to EC_50_ values obtained from these experimental findings, TFAB002s demonstrated approximately tenfold higher efficacy in inducing malignant B lymphocyte death *in vitro* [31]. The CD3/CD20 bsAbs mentioned above exhibit enhanced cytotoxicity as indicated by lower EC_50_ values, which may concurrently heighten adverse reactions in the bloodstream. The studies conducted on GB261, a CD3/CD20 bispecific antibody with an ultralow affinity to CD3, have demonstrated its remarkable ability to induce T cells for the elimination of tumor cells, achieving a TOP value of approximately 80% [32]. Furthermore, its killing curve EC_50_ value was an order of magnitude greater than that of TFAB002s, highlighting a favorable balance between safety and efficacy. Notably, during the phase I/II study for GB261, this bsAb exhibited an exceptionally advantageous safety-to-efficacy ratio [33]. Particularly noteworthy is the excellent safety profile observed for CRS, which was mild and transiently infrequent. Moreover, treatment with GB261 resulted in early-onset responses characterized by profound and enduring effects. Our findings revealed that TFAB002s effectively lysed lymphoma B cells while demonstrating moderate increases in key cytokines such as IL-2, TNF-α, and IFN-γ. Notably, cytokines associated with inflammation and immunosuppression, including IL-6 and IL-10, exhibited lower elevations. Particularly during clinical events, patients experiencing severe CRS show up to a 300-fold increase in IL-6 levels [34]. These results highlighted the improved safety profile of our approach. Moreover, the tumor volume change curve exhibited a significant downward trend while concurrently maintaining body weight, thereby confirming the effective inhibition of tumor growth by TFAB002s in this mouse model without any notable adverse effects.

CD20 is overexpressed in malignant B cells and plays a crucial role in blood lymphoma B cells, making it the most promising immunotherapeutic target for BCL [7]. TFAB002s bind to both CD20-expressing tumor cells and CD3ε-expressing T cells, causing crosslinking between T cells and tumor cells, which leads to T-cell activation, tumor cell lysis, and subsequent secretion of cytokines. In this study, TFAB002s demonstrated robust T cell activation and induced lysis of lymphoma B cells, which strictly depend on the presence of CD20 expression and the concurrent binding (cross-linking) between T cells and CD20-expressing tumor cells. Furthermore, there is a limited correlation between the activity of TFAB002s and varying cell lines exhibiting differential levels of CD20 expression, thereby minimizing the likelihood for low-CD20-expressing lymphoma B cells to escape from TFAB002s. Downregulation of CD20 confers resistance to CD20-targeted treatments in BCL [7, 35]. Therefore, subclones with low levels of CD20 expression should also be considered viable targets for TFAB002s.

## Conclusions

Collectively, these findings provided compelling evidence that TFAB002s as a novel class of tumor-targeted bispecific antibodies had promising antitumor efficacy and acceptable safety. Thereby, for the wider application of this novel Fab-FabCH3 structure, further investigations will primarily focus on extending antibody half-life, mitigating cytokine release syndrome, and exploring alternative antigen targets.

## Supporting information

S1 Fig TFAB002s did not exhibit binding affinity towards CHO-K1 cells. FACS was performed to assess TFAB002s’ binding ability towards antigen-negative parent cell line CHO-K1.
(TIF)

## References

[1] Shankland KR, Armitage JO, Hancock BW. Non-Hodgkin lymphoma. Lancet. 2012;380(9844):848–57. Epub 2012/07/28. doi: 10.1016/S0140-6736(12)60605-9 . 22835603

[2] Stanglmaier M, Faltin M, Ruf P, Bodenhausen A, Schröder P, Lindhofer H. Bi20 (fBTA05), a novel trifunctional bispecific antibody (anti‐CD20 × anti‐CD3), mediates efficient killing of B‐cell lymphoma cells even with very low CD20 expression levels. International Journal of Cancer. 2008;123(5):1181–9. doi: 10.1002/ijc.23626 18546289

[3] El Hussein S, Medeiros LJ, Lyapichev KA, Fang H, Jelloul FZ, Fiskus W, et al. Immunophenotypic and genomic landscape of Richter transformation diffuse large B-cell lymphoma. Pathology. 2023;55(4):514–24. Epub 2023/03/19. doi: 10.1016/j.pathol.2022.12.354 . 36933995

[4] Chmielewska N, Szyndler J. Targeting CD20 in multiple sclerosis—review of current treatment strategies. Neurol Neurochir Pol. 2023;57(3):235–42. Epub 2023/04/01. doi: 10.5603/PJNNS.a2023.0022 . 36999373

[5] Gingele S, Jacobus TL, Konen FF, Hümmert MW, Sühs KW, Schwenkenbecher P, et al. Ocrelizumab Depletes CD20⁺ T Cells in Multiple Sclerosis Patients. Cells. 2018;8(1). doi: 10.3390/cells8010012 . 30597851 PMC6356421

[6] Klein C, Jamois C, Nielsen T. Anti-CD20 treatment for B-cell malignancies: current status and future directions. Expert Opin Biol Ther. 2021;21(2):161–81. Epub 2020/09/17. doi: 10.1080/14712598.2020.1822318 . 32933335

[7] Smith MR. Rituximab (monoclonal anti-CD20 antibody): mechanisms of action and resistance. Oncogene. 2003;22(47):7359–68. Epub 2003/10/25. doi: 10.1038/sj.onc.1206939 . 14576843

[8] Teisseyre M, Cremoni M, Boyer-Suavet S, Ruetsch C, Graça D, Esnault VLM, et al. Advances in the Management of Primary Membranous Nephropathy and Rituximab-Refractory Membranous Nephropathy. Front Immunol. 2022;13:859419. doi: 10.3389/fimmu.2022.859419 . 35603210 PMC9114510

[9] Zinn S, Vazquez-Lombardi R, Zimmermann C, Sapra P, Jermutus L, Christ D. Advances in antibody-based therapy in oncology. Nature Cancer. 2023;4(2):165–80. doi: 10.1038/s43018-023-00516-z 36806801

[10] Bacac M, Fauti T, Sam J, Colombetti S, Weinzierl T, Ouaret D, et al. A Novel Carcinoembryonic Antigen T-Cell Bispecific Antibody (CEA TCB) for the Treatment of Solid Tumors. Clinical Cancer Research. 2016;22(13):3286–97. doi: 10.1158/1078-0432.CCR-15-1696 26861458

[11] Sun LL, Ellerman D, Mathieu M, Hristopoulos M, Chen X, Li Y, et al. Anti-CD20/CD3 T cell-dependent bispecific antibody for the treatment of B cell malignancies. Sci Transl Med. 2015;7(287):287ra70. doi: 10.1126/scitranslmed.aaa4802 . 25972002

[12] Thakur A, Huang M, Lum LG. Bispecific antibody based therapeutics: Strengths and challenges. Blood Rev. 2018;32(4):339–47. Epub 20180220. doi: 10.1016/j.blre.2018.02.004 . 29482895

[13] Stirling DR, Swain-Bowden MJ, Lucas AM, Carpenter AE, Cimini BA, Goodman A. CellProfiler 4: improvements in speed, utility and usability. BMC Bioinformatics. 2021;22(1):433. doi: 10.1186/s12859-021-04344-9 . 34507520 PMC8431850

[14] Wang C, Hong J, Yang Z, Zhou X, Yang Y, Kong Y, et al. Design of a Novel Fab‐Like Antibody Fragment with Enhanced Stability and Affinity for Clinical use. Small Methods. 2021;6(2). doi: 10.1002/smtd.202100966 35174992

[15] Budde LE, Assouline S, Sehn LH, Schuster SJ, Yoon S-S, Yoon DH, et al. Single-Agent Mosunetuzumab Shows Durable Complete Responses in Patients With Relapsed or Refractory B-Cell Lymphomas: Phase I Dose-Escalation Study. Journal of Clinical Oncology. 2022;40(5):481–91. doi: 10.1200/JCO.21.00931 34914545 PMC8824395

[16] Allen C, Zeidan AM, Bewersdorf JP. BiTEs, DARTS, BiKEs and TriKEs—Are Antibody Based Therapies Changing the Future Treatment of AML? Life. 2021;11(6). doi: 10.3390/life11060465 . 34071099 PMC8224808

[17] Li H, Er Saw P, Song E. Challenges and strategies for next-generation bispecific antibody-based antitumor therapeutics. Cellular & Molecular Immunology. 2020;17(5):451–61. doi: 10.1038/s41423-020-0417-8 . 32313210 PMC7193592

[18] Carter PJ, Lazar GA. Next generation antibody drugs: pursuit of the ’high-hanging fruit’. Nat Rev Drug Discov. 2018;17(3):197–223. Epub 20171201. doi: 10.1038/nrd.2017.227 . 29192287

[19] Minson A, Dickinson M. Glofitamab CD20-TCB bispecific antibody. Leuk Lymphoma. 2021;62(13):3098–108. Epub 20210715. doi: 10.1080/10428194.2021.1953016 . 34263696

[20] Georges GJ, Dengl S, Bujotzek A, Hesse F, Fischer JAA, Gartner A, et al. The Contorsbody, an antibody format for agonism: Design, structure, and function. Comput Struct Biotechnol J. 2020;18:1210–20. Epub 20200514. doi: 10.1016/j.csbj.2020.05.007 . 32542107 PMC7283085

[21] Dickopf S, Georges GJ, Brinkmann U. Format and geometries matter: Structure-based design defines the functionality of bispecific antibodies. Comput Struct Biotechnol J. 2020;18:1221–7. Epub 20200514. doi: 10.1016/j.csbj.2020.05.006 . 32542108 PMC7283971

[22] Labrijn AF, Janmaat ML, Reichert JM, Parren P. Bispecific antibodies: a mechanistic review of the pipeline. Nat Rev Drug Discov. 2019;18(8):585–608. doi: 10.1038/s41573-019-0028-1 . 31175342

[23] Chiu ML, Goulet DR, Teplyakov A, Gilliland GL. Antibody Structure and Function: The Basis for Engineering Therapeutics. Antibodies. 2019;8(4). doi: 10.3390/antib8040055 31816964 PMC6963682

[24] Zhao C, Zhang W, Gong G, Xie L, Wang MW, Hu Y. A new approach to produce IgG(4)-like bispecific antibodies. Sci Rep. 2021;11(1):18630. doi: 10.1038/s41598-021-97393-2 . 34545109 PMC8452627

[25] Dall’Acqua WF, Woods RM, Ward ES, Palaszynski SR, Patel NK, Brewah YA, et al. Increasing the affinity of a human IgG1 for the neonatal Fc receptor: biological consequences. J Immunol. 2002;169(9):5171–80. doi: 10.4049/jimmunol.169.9.5171 . 12391234

[26] Santich BH, Park JA, Tran H, Guo HF, Huse M, Cheung NV. Interdomain spacing and spatial configuration drive the potency of IgG-[L]-scFv T cell bispecific antibodies. Sci Transl Med. 2020;12(534). doi: 10.1126/scitranslmed.aax1315 . 32161106 PMC7437947

[27] Clynes RA, Desjarlais JR. Redirected T Cell Cytotoxicity in Cancer Therapy. Annual Review of Medicine. 2019;70(1):437–50. doi: 10.1146/annurev-med-062617-035821 30379598

[28] Basu R, Huse M. Mechanical Communication at the Immunological Synapse. Trends Cell Biol. 2017;27(4):241–54. doi: 10.1016/j.tcb.2016.10.005 . 27986534 PMC5367987

[29] Li J, Piskol R, Ybarra R, Chen YJ, Li J, Slaga D, et al. CD3 bispecific antibody-induced cytokine release is dispensable for cytotoxic T cell activity. Sci Transl Med. 2019;11(508). Epub 2019/09/06. doi: 10.1126/scitranslmed.aax8861 . 31484792

[30] Engelberts PJ, Hiemstra IH, de Jong B, Schuurhuis DH, Meesters J, Beltran Hernandez I, et al. DuoBody-CD3xCD20 induces potent T-cell-mediated killing of malignant B cells in preclinical models and provides opportunities for subcutaneous dosing. EBioMedicine. 2020;52:102625. Epub 20200123. doi: 10.1016/j.ebiom.2019.102625 . 31981978 PMC6992935

[31] Bacac M, Colombetti S, Herter S, Sam J, Perro M, Chen S, et al. CD20-TCB with Obinutuzumab Pretreatment as Next-Generation Treatment of Hematologic Malignancies. Clin Cancer Res. 2018;24(19):4785–97. Epub 20180501. doi: 10.1158/1078-0432.CCR-18-0455 . 29716920

[32] Cai W, Dong J, Gallolu Kankanamalage S, Titong A, Shi J, Jia Z, et al. Biological activity validation of a computationally designed Rituximab/CD3 T cell engager targeting CD20+ cancers with multiple mechanisms of action. Antib Ther. 2021;4(4):228–41. doi: 10.1093/abt/tbab024 . 34805746 PMC8597964

[33] Song Y, Li Z, Li L, Qian Z, Zhou K, Fan L, et al. GB261, an Fc-Function Enabled and CD3 Affinity De-Tuned CD20/CD3 Bispecific Antibody, Demonstrated a Highly Advantageous Safety/Efficacy Balance in an Ongoing First-in-Human Dose-Escalation Study in Patients with Relapsed/Refractory Non-Hodgkin Lymphoma. Blood. 2023;142(Supplement 1):1719-. doi: 10.1182/blood-2023-188444

[34] Hu Y, Sun J, Wu Z, Yu J, Cui Q, Pu C, et al. Predominant cerebral cytokine release syndrome in CD19-directed chimeric antigen receptor-modified T cell therapy. J Hematol Oncol. 2016;9(1):70. doi: 10.1186/s13045-016-0299-5 . 27526682 PMC4986179

[35] van der Horst HJ, de Jonge AV, Hiemstra IH, Gelderloos AT, Berry DRAI, Hijmering NJ, et al. Epcoritamab induces potent anti-tumor activity against malignant B-cells from patients with DLBCL, FL and MCL, irrespective of prior CD20 monoclonal antibody treatment. Blood Cancer Journal. 2021;11(2). doi: 10.1038/s41408-021-00430-6 33602901 PMC7892878

