# Supplementary figures and images for: TFAB002s, novel CD20-targeting T cell-dependent bispecific Fab-FabCH3 antibodies, exhibit potent antitumor efficacy against malignant B-cell lymphoma
Source: PLoS One. 2024 Sep 25;19(9):e0310889. doi: 10.1371/journal.pone.0310889 (PMC11423992; doi:10.1371/journal.pone.0310889)

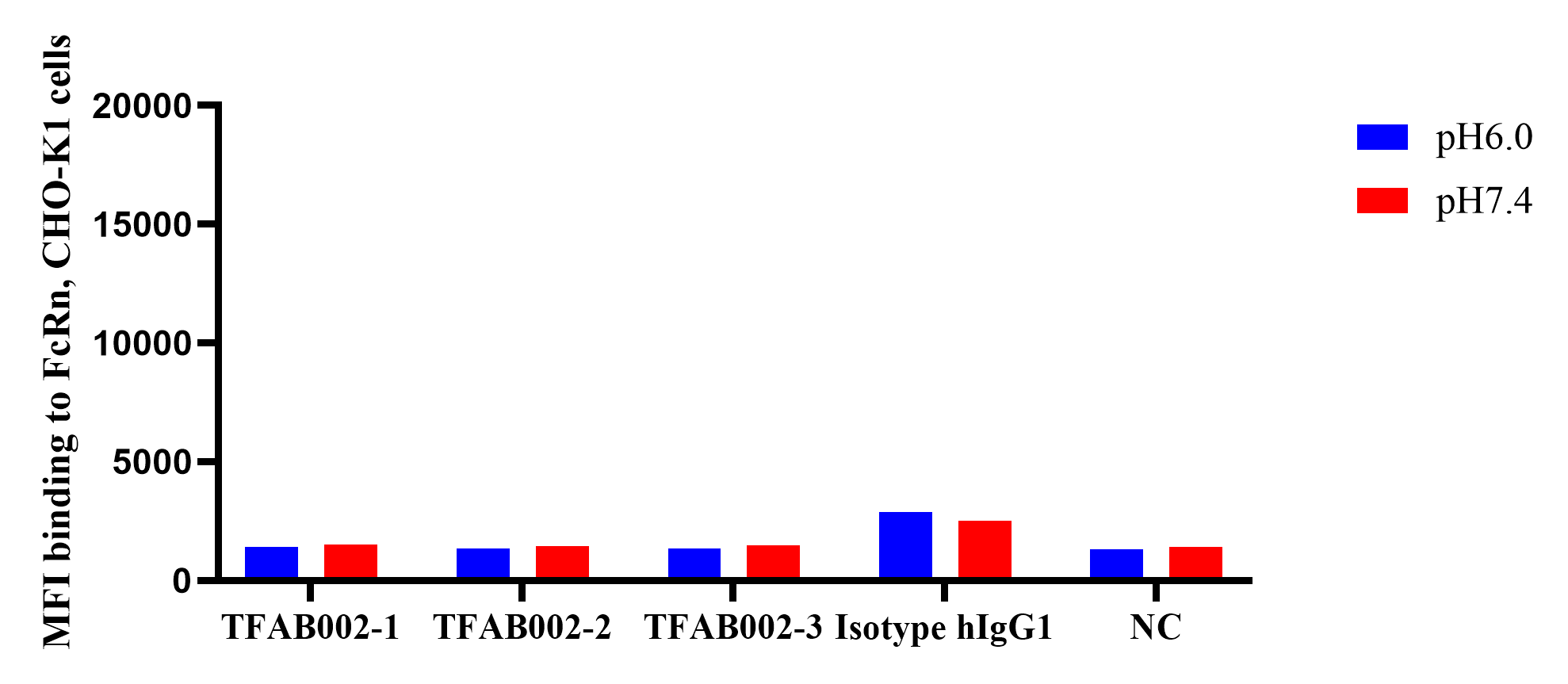

Supplement: S1 Fig — FACS was performed to assess TFAB002s’ binding ability towards antigen-negative parent cell line CHO-K1. (TIF) [file pone.0310889.s001.tif]
